# *Trichomonas vaginalis* Drug Targets and Their Role in Drug Discovery and Development

**DOI:** 10.1007/s11095-026-04098-0

**Published:** 2026-05-05

**Authors:** Mirna Samara Dié Alves, Ângela Sena-Lopes, Luiza Domingues Moron, Bárbara da Rocha Fonseca, Sibele Borsuk

**Affiliations:** Laboratório de Biotecnologia Infecto-Parasitária, Centro de Desenvolvimento Tecnológico, Biotecnologia, Federal University of Pelotas - UFPel, Campus Universitário s/n, Prédio 19, Pelotas, RS 96010–900 Brazil

**Keywords:** target deconvolution, target-based development, therapeutic targets

## Abstract

*Trichomonas vaginalis* is the causative agent of trichomoniasis, the most common and prevalent sexually transmitted infection (STI) globally, with about 156 million cases annually. Trichomoniasis is a critical public health problem, and it is aggravated due to its association with a higher risk of HIV-1 acquisition and transmission and complications such as preterm delivery and pelvic inflammatory disease. This STI is treated mainly through the 5-nitroimidazole class, specifically metronidazole and tinidazole. However, drug resistance, which can represent between 5 and 20% of clinical cases, and hypersensitivity reactions are a general concern. In this context, drug development for trichomoniasis is an ever-growing research field. Therefore, considering how important drug targets and the mechanism of action of compounds can be to drug discovery, there is a growing interest in better understanding how some molecules can be used as targets. This article offers an overview of *T. vaginalis* drug targets, their significance in metabolism, pathogenesis, or survival, and their contribution to drug development for trichomoniasis.

**Supplementary Information:**

The online version contains supplementary material available at 10.1007/s11095-026-04098-0.

## Introduction

The protozoan parasite *Trichomonas vaginalis* is the causative agent of trichomoniasis, the most prevalent and frequent non-viral sexually transmitted infection (STI) worldwide. The last estimate by the World Health Organization reported a global prevalence of 5.3% in women and 0.6% in men in 2016, with 156 million new cases in the same year [1]. In women, trichomoniasis can range from asymptomatic to severe, with the most common symptoms being odorous vaginal discharge and vaginal pruritus. In men, on the other hand, the infection is usually asymptomatic and self-limited; however, in symptomatic cases, it can cause urethritis and prostatitis [2, 3].

*T. vaginalis* is a public health concern on its own; however, its significance becomes even more pronounced due to the influence it has on increasing the risk of acquiring HIV by 1.5 to 2.7-fold [4–7]. Chemotherapeutic treatment options are mostly limited to the 5-nitroimidazole drugs, specifically metronidazole (MTZ) and tinidazole (TNZ). Although MTZ and TNZ present reliable treatment rates, there is a rising concern about resistance-related problems when considering that clinical resistance rates can represent about 10% of the cases in certain parts of the world [8–10]. In this scenario, alternative treatments are essential, and high-quality drug development studies are constantly being published [11–15].

Understanding, at least partially, the mechanism of action through which new drugs lead to parasites’ death is a critical step for proposing these molecules as trichomonacidal agents. In line with that, in the last decade, several drug targets have been proposed for *T. vaginalis*, as is the case for the 20S proteasome [13] and the enzymes thioredoxin reductase [16] and triosephosphate isomerase [17]. Also, the overall understanding of previously described targets, such as the hydrogenosome and its redox pathways [18–21], has greatly increased.

As new chemotherapeutic targets are described, their roles and applicability in the search for antitrichomonal molecules need to be investigated and better identified. An extensive array of well-established drug targets may increase target-oriented drug testing and eventually shift how drug development for trichomoniasis is accomplished. This review offers an overview of the drug targets described for *T. vaginalis* and how they are significant for developing novel alternatives for treating trichomoniasis. *T. vaginalis* pathogenesis, current treatment options and regimens, and promising compounds will also be discussed.

### *Trichomonas vaginalis*

*Trichomonas vaginalis* is a flagellated anaerobic/microaerophilic protozoan parasite that targets the human genital tract, causing trichomoniasis. *T. vaginalis* belongs to the family Trichomonadea of phylum Parabasalia [22], and it is the only trichomonad species for which pathogenicity toward humans is well-established [23]. The parasite is characterized by four anterior flagella, an undulating membrane incorporating a fifth flagellum in its outer edge, and an axostyle extending from the anterior to the posterior axis and into the extracellular environment [24, 25].

*T. vaginalis* is mostly said to have one well-characterized pleomorphic life form, the trophozoites, which are typically pyriform; however, depending on the physicochemical conditions of the environment, they can undergo morphological changes. As it happens, for instance, for its ameboid form, which is frequently seen when cytoadherence to epithelial cells is underway [26–28].

On that note, recent studies [29–31] have brought attention to a controversial *T. vaginalis* form widely known as pseudocysts, or more recently, cyst-like structures (CLS) [31] and described as non-motile, round-shaped structures without a true cyst wall and with flagella internalized in endocytic vesicles [32]. These studies characterized the ability of CLS to survive in adverse environments and highlighted their relevance in clinical infections, pathogenesis, and the parasite’s life cycle, proposing that this life form might be more important than previously acknowledged [29–31]. Their findings also raised questions about the significance of these forms for drug development against *T. vaginalis*.

Another essential characteristic of *T. vaginalis* is the lack of mitochondria, which are replaced by hydrogenosomes, hydrogen-producing organelles responsible for energy production, and several other catalytic pathways. The hydrogenosomes are mitochondria-derived organelles [33, 34]; however, there are notable characteristics that differentiate these two structures, for instance, the lack of specific DNA and cytochromes in the hydrogenosomes, which also possess a different proteome from that of mitochondria [35–37]. Due to its fundamental differences from the host’s mitochondria and its significance to biological processes in *T. vaginalis*, the hydrogenosome and its metabolic pathways are considered significant drug targets for drug development.

*T. vaginalis* has a 160 Mb haploid genome that encodes about 60,000 protein-coding genes, has a low number of introns, and is highly repetitive with repetitive elements, such as transposable elements and virally derived repeats, accounting for about half of its size [38–40]. This unusually large and highly repetitive genome can be a challenge in molecular genetic studies, but recent papers have been trying to analyze and understand how gene expression is regulated and find genetic markers for drug resistance [39, 41]. In addition, many *T. vaginalis* enzymes, such as flavin reductase [42] and superoxide dismutase [37, 43], are encoded by multiple alleles. Although it is still unclear why this happens, it certainly influences choosing certain proteins as drug targets even more when considering drug specificity to different isoforms.

This parasite’s life cycle is monoxenous, with humans as the single host. The trophozoites are said to be the only infective form; however, a recent study has shown that CLS are present in samples from vaginal swabs of infected women, suggesting they may play a role in transmission and pathogenesis [31]. *T. vaginalis* multiplies asexually through longitudinal binary fission and can be found in epithelial cells of the vagina, cervix, urethra, and prostate, where it establishes the infection.

### Cellular Pathogenesis

Transmission happens almost entirely through unprotected sexual contact, and although neonatal transmission [44] and transmission via fomites [45] have been reported, they are relatively rare. *T. vaginalis* pathogenesis is a multi-step, complex process that is still not fully understood but has been recently gaining well-deserved attention (reviewed in detail by [46, 47]). As an extracellular pathogen, once transmission occurs, cytoadherence must take place to allow the establishment of infection. Adherence to epithelial cells is multifactorial and, among other things, it depends on (i) morphological adaptation to the ameboid form; (ii) an increase in vaginal pH, which is a consequence of eliminating organisms, such as *Lactobacillus* spp., from the host’s microbiome [48]; (iii) and the activity of mucinases, adhesins, cysteine proteases (CPs) [46, 49], *T. vaginalis* (*Tv*) membrane proteins *Tv*BAP1 and *Tv*BAP2 [50], and *T. vaginalis* surface lipoglycan (*Tv*LG) [51, 52], among other molecules and mechanisms.

*Tv*LG is the most abundant surface molecule of *T. vaginalis* and binds to galectin-1 and −3 receptors in the host cells. Besides participating actively in cell adhesion, *Tv*LG modulates inflammatory responses and seems to be involved in inducing IL-8 production and macrophage inflammatory protein 3α [52–54]. Adhesin proteins (APs) are also essential to adhesion, and five of them (AP23, AP33, AP51, AP65, and AP120) have been described as important for establishing infection. Interestingly, these metabolic enzymes are mainly found in the hydrogenosomes and are described as multifunctional molecules [46, 55].

*T. vaginalis* pathogenic interaction with host cells goes beyond simply adhering to the epithelium; it also includes, among other things, the shedding of extracellular vesicles that interact with the host tissue and contain molecules that are important to promote infection [56] and a cytotoxic process that leads to damage to the plasma membrane and is mediated by CPs [57–59]. CPs significantly influence adherence [60] and immune evasion [61–63]. Considering how important CPs are to *T. vaginalis* pathogenesis, they are classified as virulence factors and potential drug targets.

Once parasites adhere to host cells, several other mechanisms take place and lead to cell death, which seems to happen both through necrosis and apoptosis [64, 65]. A few studies have described molecules that actively participate in cell destruction, as is the case for (i) *Tv*ROM1, a protease that leads to four times more host cell lysis when overexpressed [66]; (ii) *Tv*CP2, a CP that promotes cytotoxicity and cell death to host cells [65]; and (iii) *Tv*MP50, a metalloproteinase that, when inhibited, leads to a reduction in host cell death [67]. Inflammatory processes and neutrophil recruitment also take place [47].

### Trichomoniasis

Once the infection is established, clinical manifestations of the disease vary greatly. Symptoms can take from days to months to develop, and in many cases, mostly in men, the infection is asymptomatic. In women, parasites usually colonize the vaginal epithelium, the urethra, and the cervix, and common clinical manifestations include vaginal discharge and discomfort, vulvar irritation, itching, dysuria, and dyspareunia. In men, trichomoniasis is less understood and characterized, but it can lead to prostatitis [68], epididymitis [69], and reduced sperm cell motility [70].

*T. vaginalis* infection is associated with a higher risk of preterm delivery and bad pregnancy outcomes, such as low birth weight and small for gestational age infants [71–73]. A connection between this STI and a higher risk for developing pelvic inflammatory disease (PID) has also been established, and HIV + women are at even higher risk for PID [71, 74, 75].

Besides the risk it offers on its own, *T. vaginalis* infection and trichomoniasis have also been described as cofactors for HIV acquisition and transmission [4, 5, 76] and a higher incidence and prevalence of herpes simplex virus type 2 (HSV-2), particularly in women [77, 78]. The interplay between *T. vaginalis* and HIV is still not fully understood; however, it is well-accepted that trichomoniasis increases HIV acquisition and transmission [4–6, 76, 79].

Trichomoniasis is a public health threat and, at the same time, is still considered a neglected STI. This happens, at least in part, because despite its incidence, prevalence, and role in increasing HIV-1 risk and poor pregnancy outcomes, in most countries, such as Brazil and the United States, no programs or guidelines have been put in place to establish surveillance, screening, and/or control of *T. vaginalis* infection [80, 81]. In addition, in many countries, reporting trichomoniasis cases is not compulsory, leading to an underestimation of incidence and prevalence, which might also influence our knowledge of the true proportion of treatment failures and their reasons.

### Current Therapeutics and Their Problems

Trichomoniasis treatment is done using 5-nitroimidazole drugs, specifically MTZ, TNZ, and now, secnidazole (SEC), the only two FDA-approved drugs that have been in use since 1963, 2004, and 2021, respectively. However, as previously mentioned, a rise in resistant strains [8, 82–84] and problems such as hypersensitivity reactions [85] have led to a more prominent search for treatment alternatives.

The Centers for Disease Control and Prevention has a guideline for treating STIs [86], and it recommends two treatments for trichomoniasis in HIV individuals: (i) a single 2 g oral dose of MTZ or TNZ, as a first choice, and (ii) 500 mg of MTZ twice a day for 7 days as an alternative. The latter is mostly used as a first choice for HIV + individuals, recurrent infections, and treatment failures.

Interestingly, some studies have suggested that the single-dose treatment [87–90] does not seem to be the most effective one. Also, regimens using non-FDA-approved drugs have been proposed and tested in different studies [91–95], but are not perceived as guidelines.

5-nitroimidazole resistance is one of the biggest concerns when it comes to trichomoniasis, even more so when there is still no other class of drugs approved for treating this STI, and cross-resistance between MTZ and TNZ has been reported [96]. Resistance rates range from 2 to 9.6% for MTZ [8, 9, 83] and from 0 to 2% for TNZ worldwide [8]. Interestingly, some studies have correlated *in vitro* and clinical resistance. One study found that 75% (9/12) of the isolates obtained from samples suspected to be from clinically resistant trichomoniasis cases were confirmed as MTZ-resistant [82]. Another study obtained 175 samples from trichomoniasis cases where MTZ treatment failed and found that 65.7% (115/175) of the isolates were resistant, with almost half of them being considered highly resistant (minimum lethal concentration ≥ 400 µg/mL) [84]. These data show that *in vitro* resistance could be predictive of clinical resistance and possibly help in choosing treatment regimens in the future.

Besides resistance, hypersensitivity reactions are another problem resulting from the exclusive use of 5-nitroimidazoles as treatment [97]. Treating patients who have had such reactions requires a desensitization regimen, and such a strategy is not always effective. In some cases, such as pregnant women, desensitization is not recommended, leaving little to no options when it comes to treating patients that could not be desensitized. Some MTZ desensitization protocols have been proposed [98–100], and alternative treatments that do not involve 5-nitroimidazoles have been applied as well [85, 93, 94].

Altogether, the above-mentioned data make clear that drug development for trichomoniasis is essential, and novel and effective compounds are urgently needed to bring an alternative for treating *T. vaginalis-*resistant isolates and 5-nitroimidazole-sensitive and allergic patients.

### Drug Development for *T. vaginalis*

Studies aiming at finding alternative treatments for trichomoniasis have been extensively performed for years, and several compounds have been analyzed *in vitro*, *in vivo*, and in clinical settings. A recent study has reviewed chemotherapeutic options available for treating trichomoniasis [101] and, therefore, this review will briefly mention some of the most promising *in vitro*-tested compounds and a few of the options that have been, or still are, applied in clinical cases to contextualize the drug candidates that are currently in the spotlight.

Among the *in vitro*-tested compounds are auranofin [16], disulfiram [102], miltefosine [103], *N*-chlorotaurine [104], and proton pump inhibitors [105]. These are reprofiled drugs that have shown a relevant anti-*T. vaginalis* activity. Some synthetic compounds, nitrothiazole–NSAID chimeras [106], chlorinated metronidazole compounds [107], and gold(I) complexes [14] also had great trichomonacidal activities *in vitro*. As for compounds that have been tested in clinical trials, acetarsol [108], boric acid [93], paromomycin [91, 92], and pentamycin [109] had some of the best results.

Although numerous molecules are under investigation, none have been definitively approved for treating trichomoniasis. Therefore, there is still no official alternative for the treatment of *T. vaginalis*. In this sense, drug discovery studies are still underway and should remain so until the FDA approves at least one new class of drugs for treating trichomoniasis.

### Drug Discovery Approaches

Drug discovery for small molecules, either natural or synthetic, can be divided into two main approaches: phenotype-based screening and target-based screening [110]. The former is a strategy that does not require a prior understanding of a compound’s mechanism of action or molecular target. It is based on biochemical or molecular function assays, and it starts with assays to analyze compounds’ activity over pathogens or cells of interest and can continue with the identification of possible drug targets, also known as target deconvolution [110–112]. This has been the main strategy applied in antiparasitic drug discovery, and it has successfully led to biologically active compounds, many of which naturally interact with two or more parasite molecules simultaneously, a concept known as polypharmacology [113].

On the other hand, target-based screening follows the opposite direction, going from target to phenotype. It starts from a validated target molecule, or with the selection and validation of a target, for which compounds are usually selected based on results from high-throughput screening, and then to validate the desired biological activities, assays that evaluate the cellular effects of candidate drugs need to be performed [110, 114]. This strategy has been in evidence for some years in the attempt to find effective candidates for treating several diseases, and in recent years has been applied to drug development against *T. vaginalis*.

Due to the growing interest in target-based drug discovery and in defining possible mechanisms of action for valuable drug candidates, *T. vaginalis’* potential drug targets are in the spotlight. In this sense, we offer an overview of such targets and evaluate them as drug candidates based on their functions in *T. vaginalis*.

### Hydrogenosomes

When it comes to the trichomonads, the hydrogenosome was first described in *T. foetus* [115] and a couple of years later in *T. vaginalis* [116] through the characterization of the activity of several enzymes, such as hydrogenase, pyruvate synthase, and malate dehydrogenase. From its first characterization until now, the hydrogenosome has been a target of many studies, and it is currently described as a double-membrane organelle that derives from a common ancestor of the mitochondria. This organelle is essential for several catabolic processes that culminate in energy production and excretion of molecular hydrogen, and it participates in maintaining redox balance [33–36, 117].

Although hydrogenosomes and mitochondria are derived from a common ancestor, there are several differences between these two organelles. Hydrogenosomes do not possess the exclusive organellar genome and the membranous respiratory chain, which are found in mitochondria. Interestingly, the hydrogenosomal proteome was described as highly reduced, in comparison to that of the mitochondria, and at the same time extremely redundant, following the same profile seen for the *T. vaginalis* genome, with multiple paralogs of proteins [36]. For instance, there are 15 genes encoding malic enzymes, and 7 isoforms of this enzyme are found in the proteome, and the same is true for other enzymes. Although highly redundant, the hydrogenosome is a much more complex organelle than initially thought [36].

Another significant difference between these two organelles is their metabolic pathways, which differ greatly. Such disparities are partially why the hydrogenosomes have been considered effective drug targets for new compounds against *T. vaginalis* [18, 117]. It is worth noting that when hydrogenosomes are described as an excellent drug target, it usually entails their enzymes and metabolic pathways, not necessarily the organelle itself. For instance, hydrogenosomal enzymes, such as hydrogenase and pyruvate-ferredoxin oxidoreductase (PFOR) [18], are seen as appropriate targets for new drugs, either because they are not found in host cells or because their sequences/structures are not conserved. Some studies have described compounds that affect the hydrogenosome or its enzymes.

A study evaluated the effect of the treatment of trophozoites with methyl jasmonate (MJ), a stress hormone from plants, and observed that it led to the loss of hydrogenosomal membrane potential, with hydrogenosomes showing a low electron density and changes in morphology and location of these organelles inside the cell. Based on these results, the authors suggested that MJ caused irreversible damage to the hydrogenosomes, which might have contributed to parasite death [118]. Another study reported that treating *T. vaginalis* with 50 µM resveratrol (RESV) induced parasite death, reduced the enzymatic activity of a *T. vaginalis* hydrogenase (*Tv*hyd) without altering its gene expression, upregulated gene expression for PFOR, caused overexpression of hydrogenosomal Hsp70, and inhibited H_2_ production by the hydrogenosome. Taking these data into consideration, the authors suggested that RESV targets the hydrogenosome metabolism to exert its anti-*T. vaginalis* activity [18]. In addition, ultrastructural effects (membrane blebbing and disruption, cell clusters, and wrinkled cells) and changes in intracellular structures were described in trophozoites treated with methyltransferase inhibitors. Among these effects, the authors described that hydrogenosomes’ structures were abnormal and damaged [119], reinforcing hydrogenosomes as drug targets.

A recent study analyzed how curcumin (CUR) treatment affected *T. vaginalis* viability and metabolism and demonstrated that CUR (100 µM) almost doubled PFOR gene expression in *T. vaginalis* trophozoites and slightly reduced hydrogenosomal membrane potential. Also, CUR (50 µM) significantly reduced trophozoites’ general proteolytic activity [19]. These results show that CUR targets, at least partially, the hydrogenosome metabolism. A newly published study evaluated the impacts of three drugs against *T. vaginalis*, and two of them led to hydrogenosomal alterations, among other described effects. More specifically, one drug (amiodarone) limited hydrogenosomes’ localization to the cell periphery and induced an elongated shape, while the other (dronedarone) caused hydrogenosomes to present an emptied matrix [120].

It is worth mentioning that hydrogenosomal metabolism and function, for instance, protein import and amino acid catabolism, are still not fully understood, and there is a lot to be uncovered about this organelle. Thankfully, over the past decade, valuable studies have been conducted to characterize *T. vaginalis* hydrogenosomes [121–124], allowing for further discoveries in drug development and other research areas.

Altogether, these studies show how the hydrogenosome can be implicated in the suggested mechanism of action for different molecules with anti-*T. vaginalis* activity and encourage its use as a drug target for antitrichomonal agents. Interestingly, the complexity of the hydrogenosome as an organelle entails several hydrogenosomal metabolic pathways, and the molecules that participate in them can also be seen as drug targets. This is true for *T. vaginalis* redox pathways and some of their enzymes, but because these are not restricted to the hydrogenosome, they will be discussed next in separate topics.

### Redox Pathways

As anaerobic/microaerophilic organisms, one of the challenges for *T. vaginalis* survival is the reactive species of oxygen (ROS) and nitrogen (RNS) they encounter either in the environment they inhabit or as a defense mechanism put in place by the host immune system, mainly macrophages, and neutrophils [122, 125, 126]. This matters even more when considering that *T. vaginalis* redox metabolism, as for other microaerophilic organisms, depends on enzymes that are susceptible to moderate and high oxygen concentrations and, therefore, need an efficient antioxidant system to survive. In that sense, oxidative stress (enforced by ROS), nitrosative stress (brought about by nitric oxide (NO) and other RNS), and molecular oxygen (O_2_) are toxic to these parasites, and to ensure survival these pathogens need to maintain redox balance by making use of several redox pathways to eliminate both endogenous and exogenous (environment and host defense mechanisms) toxic molecules [20, 126, 127].

Several redox pathways found both in the cytosol and the hydrogenosomes constitute *T. vaginalis* metabolism, and there are different enzymes involved in ROS, NO, RNS, and molecular oxygen removal. A recent study reviewed the main aspects of the redox system in microaerophilic pathogens, the participation of redox enzymes in maintaining parasite survival, and their possible use as drug targets [20]. Therefore, we will overview the most significant aspects relating specifically to *T. vaginalis* and its redox enzymes as possible drug targets.

**Fig. 1:**
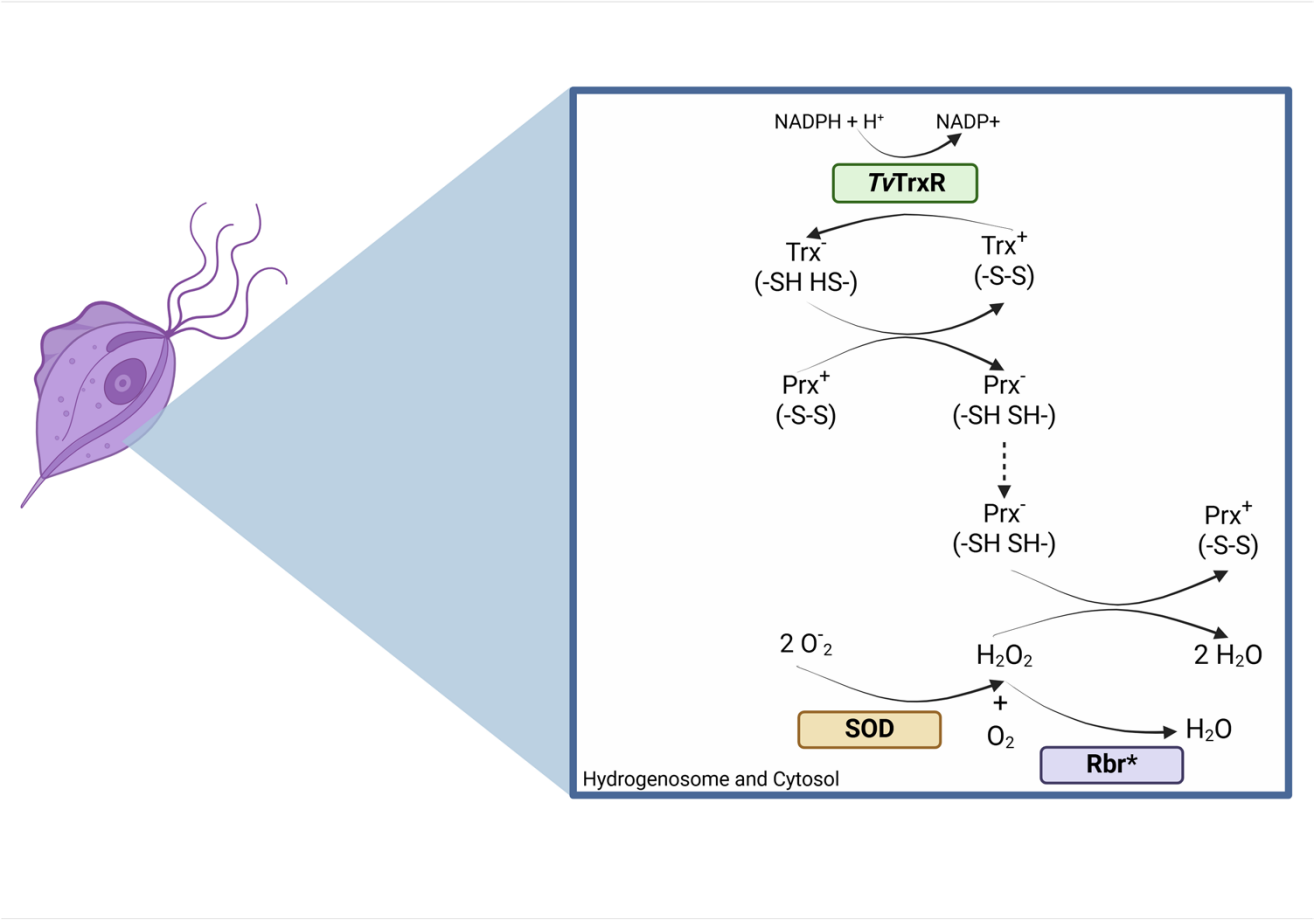
Partial redox pathway in *T. vaginalis* highlighting *Tv*TrxR function. *Tv*TrxR: *Trichomonas vaginalis* thioredoxin reductase; SOD: superoxide dismutase; Rbr: rubrerythrin. *Rubrerythrin is only found in the hydrogenosome. Created in BioRender. Dié Alves, M. S. (2026) https://BioRender.com/yeperqd.

Numerous enzymes are involved in the removal of harmful molecules from *T. vaginalis* trophozoites, for instance, superoxide dismutase (O_2_^−^ removal); peroxiredoxins (H_2_O_2_ removal); thioredoxins (peroxiredoxins recycling, H_2_O_2_ removal); functional rubrerythrin (H_2_O_2_ reduction and removal); thioredoxin reductase (thioredoxin and ribonucleotide reductase reduction, H_2_O_2_ removal, oxidized proteins maintenance and repair, excess cysteine catabolism) (Fig. 1); flavodiiron protein and flavin-dependent NADH oxidase (O_2_ reduction and removal); and flavin reductase (reduction of O_2_ to H_2_O_2_) [20, 128, 129]. In addition, cysteine synthase plays a significant role in redox balance since cysteine is a major redox buffer in *T. vaginalis* due to the lack of glutathione [130].

Interestingly, although redox pathways have been described for years as possible drug targets for compounds biologically active against protozoan pathogens, including *Trypanosoma cruzi* [131], *Plasmodium falciparum* [132], *T. vaginalis* and microaerophilic pathogens [20, 133], only in recent years studies have begun to analyze the effect of trichomonacidal compounds on these pathways. However, the number of studies is still reduced. Nonetheless, one of the redox enzymes has been gaining more attention as a drug target in the last five years. Thioredoxin reductase has been described as a viable drug target for anti-*T. vaginalis* compounds.

### *Trichomonas vaginalis* Thioredoxin Reductase (TvTrxR)

*Tv*TrxR has been in evidence in studies about *T. vaginalis* for a few years due to its importance to these parasites’ metabolism, its involvement in drug resistance, and its potential as a drug target [14, 16, 43, 128, 134, 135]. As mentioned, *Tv*TrxR has multiple functions. Its interplay in the reduction of peroxiredoxins (*Tv*Prx) via thioredoxin (Trx) reduction (Fig. 1) has an important effect on parasites’ survival since *Tv*Prx are perceived as essential because they help maintain oxidative balance by detoxifying peroxides [20]. Besides that, the inhibition of other Trx-dependent metabolic pathways, such as the reduction of ribonucleotide reductase [129] and cysteine catabolism [136], would surely harm the parasite’s viability. Altogether, impairing TrxR functions in *T. vaginalis* trophozoites presents itself as an interesting drug development strategy, and consequently, compounds that inhibit *Tv*TrxR activity have been reported [14, 16, 43, 137].

One of the compounds that interacted with *Tv*TrxR was auranofin (AF), a reprofiled drug originally prescribed as an antirheumatic. The study demonstrated that, besides leading to *T. vaginalis* death, AF directly inhibits *Tv*TrxR reductase activity with an IC_50_ of 0.085 μM in a cell-free assay, and in *T. vaginalis* trophozoites, AF inhibited *Tv*TrxR in a dose-dependent manner. The study also demonstrated that AF induces oxidative stress in trophozoites, corroborating the hypothesis that *Tv*TrxR has a role in conserving redox balance through a TrxR-dependent antioxidant metabolism [16].

Another study analyzed gold(I) phosphine derivatives for their activity against *T. vaginalis* and evaluated whether the most promising compounds could inhibit the enzymatic activity of the two most highly expressed *Tv*TrxR isoforms (TrxRc and TrxRh2, in that order). The authors found that all tested gold compounds presented a reliable inhibitory activity against *Tv*TrxR (IC_50_ < 50 nM). Also, the inhibition of cell-free purified isoforms and the inhibition of cellular *Tv*TrxR in trophozoites showed strong correlations, and the compounds’ inhibition profile was similar in both isoforms. In addition, by correlating *Tv*TrxR inhibition and anti-*T. vaginalis* activity, the authors observed that the former is an important mechanism through which these compounds induce cell death, but there are other mechanisms involved in these compounds’ trichomonacidal activity [14].

Interestingly, a study had previously proposed an alternative mechanism of action for 5-nitroimidazole drugs that implicated *Tv*TrxR as a drug target [43]. Authors characterized the formation of covalent adducts between *Tv*TrxR and MTZ, and the same was true for eight other enzymes involved in Trx-mediated redox metabolism. Based on their findings, they suggested that *Tv*TrxR can activate 5-nitroimidazole drugs, and once activated, these compounds form adducts with *Tv*TrxR and the enzymes that are in proximity, making these proteins their targets [43]. It is important to point out that the authors did not discard the previous mechanism suggested, but they believe the damage that leads to parasites’ death cannot be exclusively explained by it.

*Tv*TrxR has been validated as a potential drug target by these studies; however, there is still a lot to be uncovered in terms of how compounds inhibit these molecules (e.g., do they form adducts as shown for 5-nitroimidazoles or interact otherwise?), and how significant this inhibition is to parasites’ death, among other things. Data thus far suggests that *Tv*TrxR was not an exclusive target for the compounds analyzed. Therefore, the search for a specific TrxR inhibitor with good selectivity toward *T. vaginalis* is still underway.

### *Trichomonas vaginalis* Triosephosphate Isomerase (TvTIM)

**Fig. 2:**
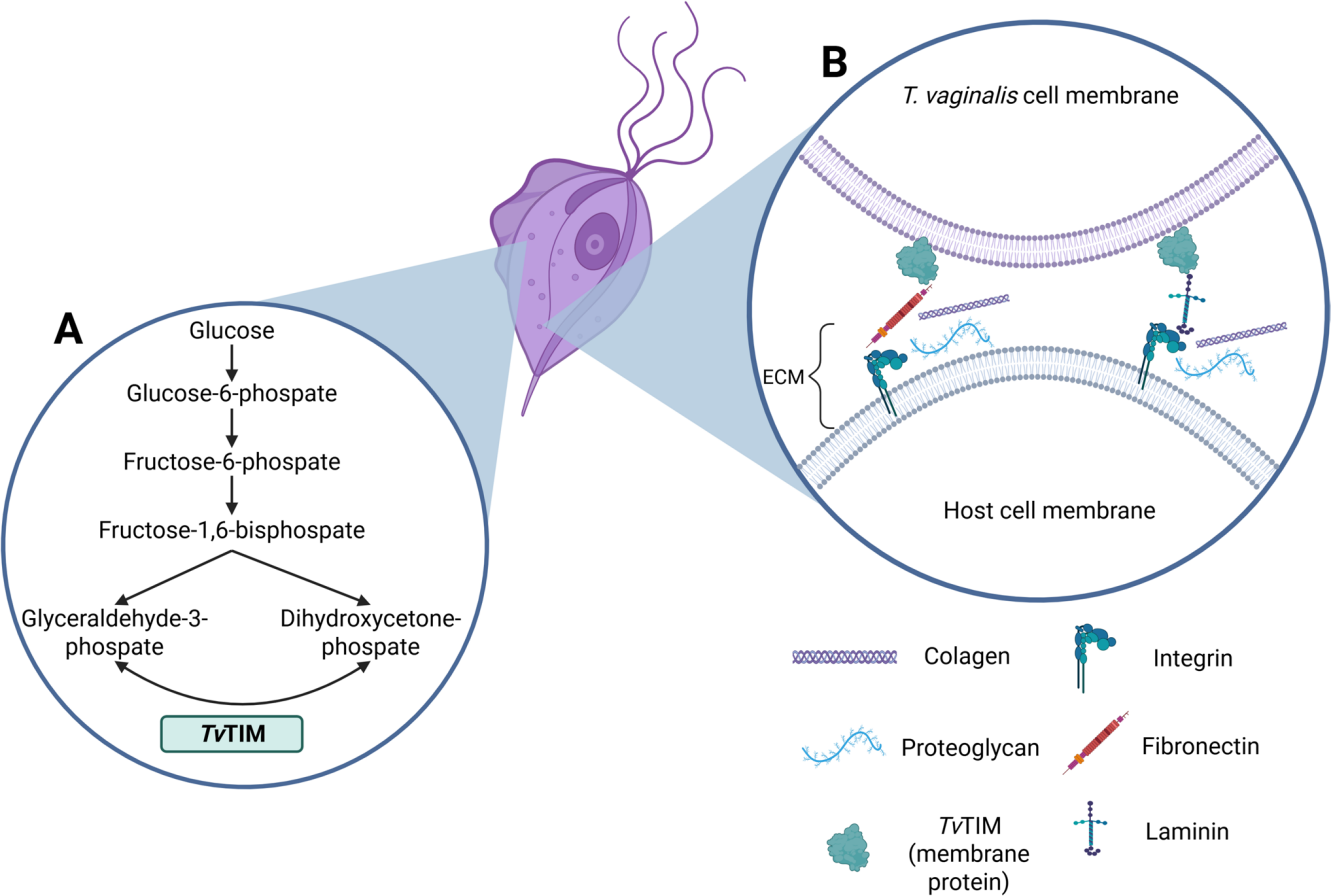
(**A**) Partial glycolysis metabolism in *T. vaginalis,* highlighting *Tv*TIM function as a glycolytic enzyme. (**B**) *Tv*TIM acts as a surface binding protein for fibronectin and laminin. *Tv*TIM: *Trichomonas vaginalis* triosephosphate isomerase. Created in BioRender. Dié Alves, M. S. (2026) https://BioRender.com/o8oi8k8.

*Tv*TIM is a glycolytic enzyme responsible for the reversible interconversion of dihydroxyacetone phosphate into glyceraldehyde 3-phosphate [138], which is part of a non-linear step of glycolysis (Fig. 2A). By catalyzing this reaction, *Tv*TIM is responsible for the net gain of glycolysis-derived ATP and for connecting the glycolytic pathway to glucogenesis, lipid metabolism, and the pentose-phosphate pathways [138]. Therefore, *Tv*TIM can be considered essential to trophozoites’ survival. Besides its glycolytic function, at least one isoform of *Tv*TIM functions as a surface protein that can bind laminin and fibronectin [139] (Fig. 2B). Therefore, studies indicated that in *T. vaginalis, Tv*TIM is a moonlighting protein and exhibits dual cellular localizations (cytosol and surface) and dual function (glycolytic enzyme and surface binding protein) (Fig. 2).

Considering its significance to the maintenance of the glycolytic pathway and how dependent amitochondriates are on glycolysis for energy, *Tv*TIM has been suggested as a drug target for *T. vaginalis* [17, 140, 141]. TIM has also been described as a drug target for other parasitic species, such as *Giardia lamblia* [142]*, Trypanosoma cruzi* [143], and *Plasmodium falciparum* [144].

Because of *T. vaginalis’* highly repetitive genome, *Tv*TIM is encoded by two functional genes (*tpi1* and *tpi2*), and the two protein isoforms that derive from them are *Tv*TIM1 and *Tv*TIM2, respectively. Despite being encoded by heterologous genes, which are almost 98% identical, *Tv*TIM1 and *Tv*TIM2 have a 98.4% identity between their amino acid sequences and only four different residues. As a result, their isoforms have common catalytic characteristics and are almost identical homodimers; however, their structural stability is directly affected by this small difference, with *Tv*TIM1 being more stable than *Tv*TIM2 [145, 146]. In addition, *Tv*TIMs only have 54% of amino acid identity with *Homo sapiens* (*Hs*) TIM. Altogether, these data show that *Tv*TIM has significant potential as a drug target in drug development, and with more information available on this enzyme and its relevant function, studies have started to analyze its applicability as a drug target.

Setzer *et al*. (2017) performed in silico analyses of more than 900 phytochemicals known to present antiprotozoal activity and analyzed their interactions with enzymes described as potential drug targets in *T. vaginalis*, including *Tv*TIM. None of the phytochemicals they investigated interacted in silico with *Tv*TIM. Nonetheless, their results did not discredit the use of *Tv*TIM as a drug target, and later studies showed this enzyme’s potential as a drug target.

A recent study applied high-throughput virtual screening to select compounds that target *Tv*TIM, the molecules with the best in silico interactions were obtained, and their effect on *Tv*TIM enzymatic activity was assayed. The authors found two potential binding sites for *Tv*TIM1 and *Tv*TIM2, and based on them, selected the 34 compounds with the best binding potentials. Although none of them completely inhibited enzymatic activity *in vitro*, three compounds selectively reduced the enzymatic activity of *Tv*TIM2, possibly due to its instability. The authors concluded by defending the application of chemical virtual libraries and computational tools in drug development and affirming that *Tv*TIM is a viable drug target [17].

Two other studies performed by the same group applied a different high-throughput virtual screening methodology to find potential *Tv*TIM inhibitors. The authors found that two compounds (A4 and D4) exhibited a trichomonacidal effect in *T. vaginalis* but did not inhibit *Tv*TIM enzymatic activity *in vitro* [141, 147]. Although compound A4 did not affect enzymatic activity, it altered the enzyme’s fluorescence emission spectra, which, according to the authors, could suggest that it may interfere with an important non-glycolytic function of the enzyme [141]. A previous finding that anti-*Tv*TIM IgGs led to the inhibition of *T. vaginalis* binding to extracellular matrix proteins [139] corroborates the authors’ hypothesis. Moreover, Benítez-Cardoza *et al*. (2022) observed in silico that A4 and D4 are more selective towards (better ΔG_binding_ energy) *Tv*TIM than *Hs*TIM, which could corroborate their non-glycolytic effect on these proteins.

Moreover, Palos *et al*. (2021) reported four compounds that were active against *T. vaginalis* and inhibited the *in vitro* enzymatic activity of *Tv*TIM. The inhibition rates varied from 31 to 50% at 50 µg/µL and 61.7% to 100% at 100 µg/µL; however, it is unclear if these concentrations are equal to or lower than the IC_50_ observed for *T. vaginalis* [148]. Nonetheless, the authors demonstrated that *Tv*TIM is a valuable drug target, and its inhibition could be the mechanism of action through which the compounds led to parasite death.

Recently, [149] demonstrated n-butyl (namely T-148) and iso-butyl (namely T-158, T-161, T-164, T-165, T-168, and T-169) quinoxaline-7-carboxylate-1,4-di-N-oxide derivatives with significant trichomonacidal activity and noteworthy in silico interactions with *Tv*TIM. Authors evaluated the compounds through molecular docking studies and described the n-butyl derivative T-148 (ΔG_binding_ −7.44 kcal/mol), and the iso-butyl derivatives T-168 and T-169 (ΔG_binding_ −7.94 kcal/mol and −7.41 kcal/mol, respectively) as the most promising *Tv*TIM inhibitors. The trichomonacidal effect for T-148 (IC_50_ = 83 nM), T-168 (IC_50_ = 150 nM), and T-169 (IC_50_ = 116 nM) was also promising. These results endorse *Tv*TIM as a viable drug target against *T. vaginalis*.

Studies applying *Tv*TIM as a drug target are recent and, in a way, limited. However, the enzyme TIM has been successfully validated as a drug target in other protozoan species, such as *G. lamblia* [150]. Such results should encourage researchers to keep studying *Tv*TIM and its application in drug development for trichomoniasis.

### *Trichomonas vaginalis* Methionine Gamma-lyase (TvMGL)

**Fig. 3:**
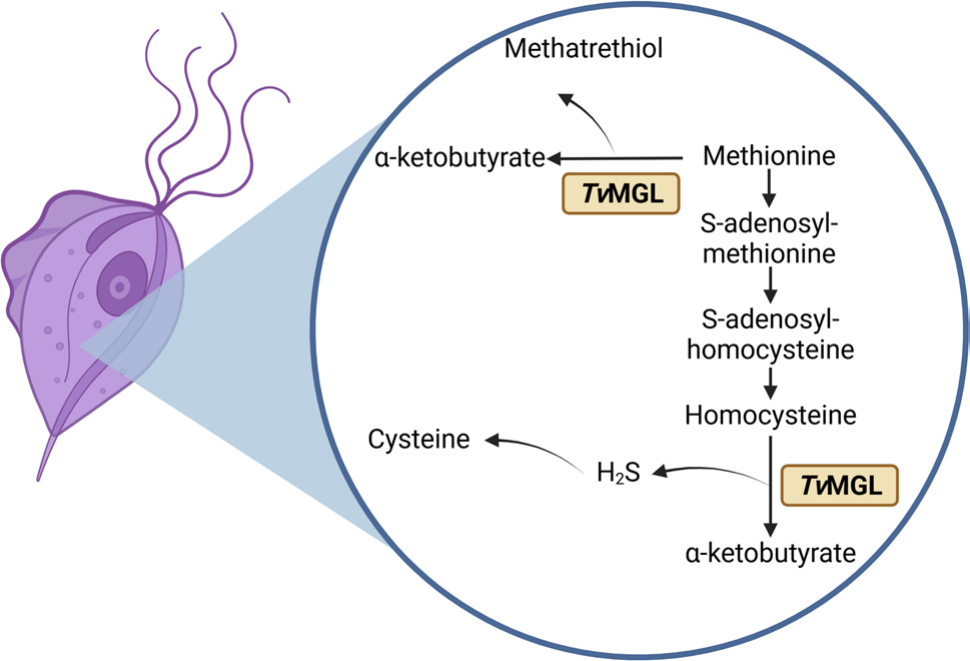
Partial sulfur amino acids metabolism in *T. vaginalis*, evidencing *Tv*MGL function. *Tv*MGL: *Trichomonas vaginalis* methionine gamma-lyase. Created in BioRender. Dié Alves, M. S. (2026) https://BioRender.com/w62pj2p.

Methionine gamma-lyase (MGL) is a pyridoxal 5’-phosphate-dependent enzyme found in a limited number of organisms, including bacteria, plants, and parasitic protozoa, but not humans. MGL is the main constituent of a unique pathway that is responsible for converting sulfur-containing amino acids (SAAs), such as methionine and cysteine, into α-keto acids, ammonia, and volatile thiols in these organisms (Fig. 3) [151]. Sulfur is essential to all organisms, and SAAs participate in several biological processes, including vitamin biosynthesis and protein synthesis [151, 152]. Besides that, in anaerobic parasitic protozoa, MGL may also be involved in energy production since one of its products (2-oxybutyrate) interacts with Coenzyme A, and this complex could be used for ATP generation [151]. The functions described for MGL corroborate with how important this pathway is for survival; add that to the fact that it is a pathway found in pathogens but absent or different in their hosts, and it becomes clear why MGL is described as a viable drug target.

*Tv*MGL consists of four identical subunits, adding up to a molecular weight of approximately 160 kDa, and resembles the MGL found in bacteria [153]. Two isoforms, *Tv*MGL1 and *Tv*MGL2, with 69% identity and different substrate specificities, have been reported for this enzyme [151, 154]. Once again, probably because of *T. vaginalis’* highly repetitive genome, this duplicity may be related to pathogenesis or metabolic functions. Interestingly, although *Tv*MGL was first purified and characterized three decades ago [153], it has remained almost virtually unexplored as a drug target for *T. vaginalis*. However, *Tv*MGL is still proposed as a potential drug target in drug development for the parasites in which it is found [140, 155–159].

To the best of our knowledge, the first work to analyze the potential of *Tv*MGL as a drug target [160] was performed ten years after its first report. The authors analyzed a prodrug, trifluoromethionine (TFMET or TFM), designed to be activated by *Tv*MGL. They confirmed TFMET was successfully activated by *Tv*MGL, generating carbonothionic difluoride, which was confirmed to be toxic to the parasite but not to mammalian cells. TFMET (5 µg/mL) led to *in vitro* inhibition of *T. vaginalis* growth, and treatment with a single dose of TFMET (40 mg/kg) was able to avoid lesions in 5 of 6 mice inoculated with *T. vaginalis*. A decade later, a study further evaluated TFMET as a *Tv*MGL inhibitor through mechanistic studies and reported another compound, L-difluoromethionine (DFM), as active against *T. vaginalis* (IC_50_ = 40.1 µM) and capable of inhibiting *Tv*MGL [161].

Three other studies suggested that *Tv*MGL might be a viable drug target for anti-*T. vaginalis* compounds based on findings from computational approaches [140, 158, 159]. The first [140] used molecular docking analyses to evaluate how several antiprotozoal phytochemicals interacted in silico with *Tv*MGL and reported that many had shown potential as *Tv*MGL inhibitors in silico, some with docking scores comparable to the co-crystallized ligand. The second [158] presented a new 2’-hydroxichalcone (3c) with anti-*T. vaginalis* activity (at 100 µM on its own and 12.5 µM when associated with MTZ) that showed in silico potential as a *Tv*MGL inhibitor (ΔGbind of − 7.8 kcal/mol and two hydrogen bonds with residues in the enzyme active site). Similarly, the third study [159] presented two compounds, PFUR 4a and PFUR 4b, with anti-*T. vaginalis* activity (MIC of 6.25 µM and IC_50_ of 1.69 µM and 1.98 µM, respectively), and one of them, PFUR 4b, showed in silico potential as a *Tv*MGL inhibitor (ΔGbind of – 5.7 kcal/mol and three hydrogen bonds with residues in the enzyme active site). Although these studies did not test the inhibitory effects of the compounds *in vitro*, they are a starting point for *Tv*MGL application and validation as a drug target in *T. vaginalis* and encourage further evaluation of its use.

### 20S Proteasome

Proteasomes are large multi-subunit protease complexes consisting of a proteolytic core (20S), and their barrel-shaped structure is formed by two rings of seven β subunits (β1 to β7) sandwiched between two rings of seven α subunits [162]. The proteasome is involved in several cellular functions, for instance, (i) degradation of damaged or misfolded proteins, short-lived regulatory proteins, and unfolded proteins; (ii) normal protein turnover; and (iii) metabolic regulation through selective degradation of transcription factors and regulatory proteins [163–165]. The catalytic subunits β1, β2, and β5 are responsible for performing these functions, and their substrate specificities vary between species [166]. A study confirmed that *T. vaginalis* has these three subunits and that they are functional [13].

Due to the proteasome’s significance, in the last few years, different studies have been exploring proteasome inhibition as a drug development strategy for antiparasitic against protozoan pathogens, such as *Leishmania donovani* [167, 168], *T. cruzi* [167, 169], *P. falciparum* [170, 171], and *T. vaginalis* [13, 172, 173].

*T. vaginalis* encodes more than 400 proteases across five classes [38] and has functional proteasome subunits, but not much is known about their significance in this parasite’s metabolism and survival. Thus far, two studies have validated *T. vaginalis* proteasome as a drug target for drug development for trichomoniasis [13, 173], and another study successfully promoted a structural elucidation of *T. vaginalis* 20S proteasome, including its binding to inhibitors [172]. In the first validation study, the authors tested different class-specific protease inhibitors against *T. vaginalis*. Although they found seven good hits for *in vitro* trichomonacidal activity (IC_50_ values equal to or lower than MTZ) and proteasome inhibition, this inhibitory activity was not as selective as desired. Considering these results, the authors decided to test molecules from the carmaphycin class, which had a potent activity against *P. falciparum* proteasome [170]. They found a molecule (carmaphycin-17 or CP-17) with significant anti-*T. vaginalis* activity (IC_50_ = 217 nM) and reduced HeLa cytotoxicity [13].

One main limitation when evaluating *T. vaginalis* 20S proteasome as a drug target is low yields and purity levels of native proteasome samples, which have directly impacted further development. However, a recent study has shown that expression of recombinant *T. vaginalis* proteasome offers a solution [172]. Authors successfully expressed a fully functional *T. vaginalis* 20S proteasome and determined its structure in complex with two known proteasome inhibitors (CP-17 and marizomib). Results showed high structural consistency when comparing native and recombinant *T. vaginalis* 20S proteasome and established a structural basis for developing specific inhibitors [172].

The most recent study used the recombinant *T. vaginalis* proteasome to evaluate the inhibitory activity of an extensive library of proteasome inhibitors, as well as newly synthesized compounds, and found many compounds capable of inhibiting *T. vaginalis* growth and survival with a > 200-fold selectivity over human cells (HeLa cell line) and low IC_50_ (10–20 nM). Authors highlighted two compounds (8 and 12) as the best proteasome inhibitors identified in their study and, most importantly, suggested that inhibiting the catalytic β5 subunit alone is enough to mediate antiparasitic effects [173].

Altogether, these findings established the *T. vaginalis* proteasome as a viable drug target for therapeutic strategies against trichomoniasis and led to a whole new class of compounds to be explored: proteasome inhibitors.

### *Trichomonas vaginalis* Drug Targets: What Should Be Considered?

This review discussed the available validation data for six proposed *T. vaginalis* drug targets; however, other molecules have been suggested as druggable targets. For instance, many cysteine proteases and metalloproteinases have been described as part of *T. vaginalis* pathogenesis [60, 62, 67, 174]; *T. vaginalis* lactate dehydrogenase (*Tv*LDH) [140, 159]; and molecules involved in the synthesis and transport of polyamines [175–178] have also been considered as targets. Table I summarizes the drug targets mentioned in this review, their biological functions, their proposed effects, and levels of experimental validation.

**Table I:** *Trichomonas vaginalis* Drug Targets: an Overview

Drug targets	Biological function	Validation studies	Validation platform(s)	Proposed/characterized effect(s) when targeted
Hydrogenosome	Energy production, molecular hydrogen excretion, and oxidative balance maintenance	[18, 19, 118–120, 179]	*In vitro* (biochemical; cell-based assay)	Loss or reduction of membrane potential; morphological and localization changes; changes in enzymes expression and H_2_ production; inhibition of hydrogenosomal enzymes (e.g., PFOR)
Redox pathways (in general)	Oxidative balance maintenance	[20, 130]	*In silico*	Key enzymes as drug targets (inhibition); oxidative imbalance leading to oxidative stress; Theoretical proposition based on other parasites
*Tv*TrxR	Thioredoxin and peroxiredoxin’s reduction and oxidative balance maintenance	[14, 16, 159]	*In silico* *In vitro* (biochemical)	In silico interactions; inhibition of *in vitro* enzymatic activity associated with oxidative stress stimulation
*Tv*TIM	Interconversion of dihydroxyacetone phosphate to glyceraldehyde 3-phosphate (Glycolysis)	[17, 140, 141, 147–149, 180]	*In silico* *In vitro* (biochemical)	In silico interactions; inhibition or reduction of *in vitro* enzymatic activity; possible inhibition of non-glycolytic activity (binding to extracellular matrix proteins)
*Tv*MGL	Energy production and conversion of sulfur-containing amino acids (vitamin and protein biosynthesis)	[140, 158–161]	*In silico* *In vitro*	Prodrug activator; in silico interactions with compounds active against *T. vaginalis*
20S Proteosome	Protein degradation and metabolic regulation	[13, 172, 173]	*In silico* *In vitro* (biochemical)	Protease inhibitors active against *T. vaginalis*; inhibition of ß1 and ß5 subunits
Metalloproteinases	Surface protease; *T. vaginalis* virulence factor; *T. vaginalis*-induced apoptosis of host cells	[67, 181–183]	*In silico* *In vitro* (biochemical; cell-based assay)	Impaired apoptosis induction in host cells (zinc-dependent cytotoxicity); proteolytic activity inhibition
Cysteine proteinases	Involved in nutrient acquisition, cytotoxicity, cytoadherence, hemolysis, and immune response evasion	[60, 174, 184–186]	*In silico* *In vitro* (biochemical; cell-based assay)	Impaired apoptosis induction (cytotoxicity) in host cells; impaired cytoadherence to host cells; inhibition of hemolysis; proteolytic activity inhibition
*Tv*LDH	Interconversion of pyruvate and lactate for ATP production	[158, 159, 187–189]	*In silico*	In silico interactions with compounds active against *T. vaginalis*; previously characterized as a drug target in other parasitic protozoans
Polyamine metabolism (synthesis and transport)	Involved in cell growth and differentiation, DNA and RNA stabilization, free radical scavenging, lipid peroxidation prevention, and pathogenesis	[175–178]	*In vitro* (biochemical; cell-based assay)	Inhibition of enzymes involved in polyamine metabolism (cytotoxic effect); inhibition of contact-dependent cytotoxicity

The table describes proposed *T. vaginalis* drug targets, their biological functions, and the current level of experimental validation. The main proposed or characterized effects upon target modulation are also included and were gathered from the studies mentioned for each target. *Tv*TrxR: *Trichomonas vaginalis* thioredoxin reductase; *Tv*TIM: *Trichomonas vaginalis* triosephosphate isomerase; *Tv*MGL: *Trichomonas vaginalis* methionine gamma-lyase; *Tv*LDH: *Trichomonas vaginalis* lactate dehydrogenase.

Selecting a drug target is a critical determinant of success in target-based drug development, and since drug resistance is a rising concern for *T. vaginalis* treatment, new targets should be chosen and designed to minimize the parasite’s ability to bypass or tolerate drug action. In other words, target selection should consider their resistance liability, defined as the likelihood that genetic or phenotypic adaptations will reduce drug efficacy under selective pressure. Although resistance liability is difficult to predict, it should be integrated early in target evaluation. Key determinants include pathway redundancy, isoform diversity, genetic and metabolic plasticity, target essentiality, and the structural tolerance of the target to resistance-conferring mutations [112, 114, 190–193].

In that sense, an important aspect when contemplating *T. vaginalis* enzymes as drug targets is how compounds interact with their isoforms. The *T. vaginalis* genome is highly repetitive and filled with paralogs, often encoding isoforms of the same enzyme. For instance, *Tv*TrxR and superoxide dismutase have five isoforms each, while *Tv*TIM and *Tv*MGL have two isoforms each, and there are at least seven isoforms for peroxiredoxins [20, 121, 145, 154]. These isoforms can have different subcellular localizations, expression patterns, and amino acid profiles, which can significantly influence drug binding and efficacy. Consequently, drug development strategies should account for this diversity, either by achieving inhibition of all isoforms or by selectively targeting the most functionally relevant one(s).

Targets in redundant pathways, such as glycolysis and general redox metabolism, may have higher resistance liability. In these systems, compensatory mechanisms or metabolic flux redistribution might mitigate the effects of enzyme inhibition [20, 112]. This is relevant for enzymes such as *Tv*LDH, for which alternative pathways may partially sustain parasite viability [38]. On the other hand, targets associated with unique or essential pathways, such as hydrogenosomal metabolism or *Tv*MGL, may present a lower resistance liability due to their limited redundancy and critical role in parasite survival [20, 133]. However, isoform diversity and adaptive changes in gene expression could still contribute to reduced drug susceptibility [121, 135].

Redox enzymes, such as *Tv*TrxR, are essential for maintaining oxidative balance, yet their integration within broader antioxidant networks may allow partial functional compensation under selective pressure [20, 133]. Similarly, pathogenesis-associated proteases, including cysteine proteinases and metalloproteinases, are often encoded by large gene families with overlapping functions [46, 47], enabling compensatory expression or functional substitution that may compromise the long-term efficacy of inhibitors targeting individual proteases.

Altogether, these considerations highlight that resistance liability should be an integral component of target prioritization in *T. vaginalis*. Incorporating this perspective early in drug discovery efforts may improve the selection of robust targets and reduce the risk of rapid resistance emergence. Targeting highly conserved, essential, non-redundant targets whose activity cannot be easily downregulated or bypassed might be a path to follow. Nonetheless, fully predicting resistance liability remains challenging, and prematurely excluding targets without thorough evaluation may lead to the loss of viable therapeutic opportunities. Achieving the appropriate balance between robustness and feasibility remains a central challenge in antiparasitic drug discovery.

Besides resistance liability, other parameters can directly influence target suitability, as is the case for selectivity, validation level, the availability of known inhibitors, and well-defined binding sites, among others [114, 193, 194]. Evaluating such parameters in early drug development might provide a more accurate assessment of a target’s potential for drug development.

In an attempt to enhance the translational significance of the data presented here for the proposed drug targets, the evidence-based overview in Table I was applied as a foundation for a prioritization framework that might help in determining which targets could be prioritized when choosing to pursue a target-based development. For that, the proposed targets were comparatively evaluated and prioritized using a qualitative framework constituted by five parameters: validation level, druggability, selectivity, resistance liability, and availability of chemical matter (Table II).

**Table II:** Prioritizing *Trichomonas vaginalis* Drug Targets Based On a Qualitative Scoring Framework

Target	Validation Level (Score)	Druggability (Score)	Selectivity (Score)	Chemical Matter (Score)	Resistance Liability (Score)	Final Score	Priority
Hydrogenosome (PFOR, hydrogenase, etc.)	Biochemical + cell-based (2)	Medium (2)	Medium–High (2–3)*	Limited (2)	High (1)	9–10	Medium
Redox pathways (general enzymes)	In silico (1)	Medium (2)	Low–Medium (1–2)*	Limited (2)	High (1)	7–8	Low-Medium
*Tv*TrxR	In silico + Biochemical (2)	High (3)	Medium (2)	Available (3)	Medium (2)	12	High
*Tv*TIM	In silico + Biochemical (2)	Medium (2)	Medium (2)	Limited (2)	Medium (2)	10	Medium
*Tv*MGL	In silico + Biochemical (2)	Medium (2)	High (3)	Limited (2)	Medium (2)	11	Medium
20S Proteasome	In silico + Biochemical + cell-based (3)	High (3)	Medium (2)	Available (3)	Medium (2)	13	High
Metalloproteinases	In silico + Biochemical + cell-based (2)	Medium (2)	Low-Medium (1–2)*	Limited (2)	Medium (2)	9–10	Medium
Cysteine proteinases	In silico + Biochemical + cell-based (2)	Low-Medium (1–2)*	Low-Medium (1–2)*	Limited (2)	High (1)	7–9	Low-Medium
*Tv*LDH	In silico (1)	Medium (2)	Low (1)	Limited (2)	High (1)	7	Low
Polyamine metabolism	Biochemical + cell-based (2)	Medium (2)	Low-Medium (1–2)*	Limited (2)	Medium–High (1–2)*	8–10	Medium

The validation level score was based on the available experimental evidence supporting target essentiality and biological relevance, with more detailed characterization/validation studies yielding higher scores. The druggability score focused on the structural and biochemical understanding of the proposed targets, while the selectivity score focused on similarity to human homologs and how likely targets were to induce parasite-specific inhibition without side effects in the host. The chemical matter score indicates how far along the drug targets are in the drug discovery pipeline, differentiating targets with no known inhibitors from those with available compounds suitable for optimization or repurposing. Lastly, the resistance liability score evaluates how likely a target is to lose efficacy over time due to resistance mechanisms. A more detailed definition of the criteria for scoring each parameter can be found in Supplementary Table 1 (Table S1). Together, these parameters enable a multidimensional and structured qualitative assessment of *T. vaginalis* drug targets, integrating biological, chemical, and translational perspectives while acknowledging the limitations and evolving nature of the available evidence.

According to the priority levels in Table II, the 20S proteasome and *Tv*TrxR are the only two high-priority targets for drug development against *Trichomonas vaginalis*. Interestingly, redox pathways were classified as low to medium priority, showing that a specific target within a pathway might be more valuable as a drug target than the pathway itself. The cysteine proteinase protein family was also classified as low to medium priority. That makes sense considering that evaluating each parameter for a specific enzyme allows a more direct score than for a pathway or protein family. Moreover, *Tv*LDH was classified as low priority due to limited validation and selectivity, and high resistance liability (compensatory pathways available). All other targets were classified as medium priority; thus, further studies are needed to better elucidate their suitability as drug targets.

Finally, the data in Table II is a qualitative tool to help guide the understanding of where current drug targets stand based on the five parameters used for evaluation. It aims at transforming the data available for each proposed target into translational information to guide possible decision-making in *T. vaginalis* drug development. Nevertheless, new data on these targets or the validation of new ones can impact this framework and shift how these targets are perceived.

Drug targets were evaluated based on five parameters relevant to drug development: (i) validation level, (ii) druggability, (iii) selectivity, (iv) availability of chemical matter, and (v) resistance liability. Each parameter was scored from 1 (least favorable) to 3 (most favorable), except for resistance liability that was inversely scored. The total score was used to classify targets into priority levels: low (≤ 7), medium (8–11), and high (12–15). This framework aims to integrate experimental evidence with translational considerations to support decision-making in drug development against *T. vaginalis*. Nonetheless, this is a qualitative tool intended to guide future research rather than provide a definitive ranking. *Score depends on the specific target being evaluated for this protein family or metabolic pathway.

### Future Perspectives on Drug Development for Trichomoniasis

The search for alternative treatments for trichomoniasis has been underway for several decades, almost since the first reports of MTZ resistance were published. However, over the last decade, several studies have helped enhance our understanding of the pathogen *T. vaginalis*. With more information on this parasite’s biology, novel virulence factors came to light, and in consequence, potential drug targets were suggested [13, 16, 17, 140]. Nevertheless, most of these targets, and for that matter, the ones we have already been aware of, are not being directly used for drug development or have only recently been gaining attention.

For years, research on anti-*T. vaginalis* compounds that can offer an alternative to 5-nitroimidazoles have occurred, without focusing on the cellular target these molecules would act on once in contact with the parasite, and, most importantly, this was not a criterion for choosing compounds. Over time, studies started to include assays that attempted to narrow down metabolic pathways in which compounds might be acting or assess their interference in cytoadherence. Assays were based mainly on physiological or phenotypical changes the trophozoites went through after treatment, for instance, changes in trophozoites’ growth and viability, *in vitro* biochemical reactions to treatment, compounds’ cytotoxicity to mammalian cells, and, more recently, altered gene expression post-treatment. Therefore, drug development against *T. vaginalis* mainly follows a phenotype-based screening profile; however, in many studies, target deconvolution is not performed.

Although target-based screening has been successfully applied to discovering drug candidates for other parasitic diseases [195], only in recent years have studies started exploring this strategy in drug discovery for trichomoniasis [13, 17, 141]. Therefore, target-based drug development for this disease is in its initial stages. In that sense, the number of targets undergoing validation or already validated for *T. vaginalis* and other amitochondriate organisms is small [155, 195], and considering the emergence of drug resistance in clinical cases, efforts should be made to further evaluate these targets.

Phenotype-based drug development provides high biological relevance, does not require previous knowledge of molecular targets, making it the best strategy for biologically complex or poorly understood organisms, and offers more advantages when target validation is incomplete or underway. However, this approach renders drug candidates with unknown mechanisms of action, leading to the need for downstream target deconvolution, which can be a complex and time-consuming process. In addition, it has a lower throughput than target-based discovery [114, 196, 197], but it has been successful in identifying drugs for several infectious and non-infectious diseases [196].

Conversely, target-based drug development allows the high-throughput screening of small molecules and offers advantages when well-characterized and biologically validated molecular targets are available. For instance, this approach works effectively when targets are essential for parasite survival, and their function is supported by genetic, biochemical, or pharmacological evidence. This strategy is also valuable in the context of emerging drug resistance because it allows the identification of alternative targets and compounds that do not rely on classical activation mechanisms [114, 193, 195–197]. Nonetheless, the predictive power of target-based approaches is hindered by parasite complexity; the activity observed against isolated targets may not directly translate into *in vivo* efficacy, and molecules from target-based approaches do not usually act through polypharmacology, which could be particularly challenging in parasitic diseases in which interaction with a singular target might be enough to guarantee parasite death [114, 193, 195–197].

Considering the limitations presented for both approaches, workflows that combine phenotype and target-based strategies might be particularly effective for antiparasitic drug discovery. Such an approach might facilitate drug discovery by integrating functional and mechanistic knowledge. These workflows typically start with phenotypic screening for the identification of biologically active compounds. Once identified, these compounds go through confirmation and prioritization steps, including dose–response analyses and counter-screening against host cells to assess selectivity, followed by target deconvolution assays [196–199]. The discovery of spiroindolones, such as KAE609 (cipargamin), followed by the elucidation of its target (PfATP4), of selective proteasome inhibitors in *Plasmodium falciparum* are two examples of how this workflow can contribute to antiparasitic drug development [200, 201].

Finally, *T. vaginalis* target validation remains challenging, often making phenotypic screening the most reliable entry point, while target-based approaches become increasingly valuable once well-characterized targets are identified. In addition, data reviewed here suggest that target-based drug discovery is a research gap in drug development against trichomoniasis and should be further explored over the next decade. Exploring target-based drug development for *T. vaginalis* might open a new set of possibilities when it comes to finding trichomonacidal drug candidates and the information provided here for the current targets. However, there is no indication that phenotype-based drug discovery will be substituted, even more so when considering that it has been a successful strategy for many infections [112, 114]. Even more when target deconvolution techniques can help with proposing mechanisms of action for compounds discovered through phenotype-based drug development [110, 111]. Besides, combining phenotypic and target-based approaches becomes a third strategy to be considered for *T. vaginalis* drug development [200, 201].

All these strategies have advantages and disadvantages, and choosing one or the other will depend on a variety of factors, including specific objectives and available resources. Nonetheless, whichever choice is made towards a drug development strategy, researchers should keep in mind that trying to establish a mechanism of action or a drug target for promising compounds is an essential step in proposing a molecule as a viable alternative treatment.

## Concluding Remarks

Treatment alternatives for trichomoniasis are in high demand since relying exclusively on 5-nitroimidazoles is not a good long-term strategy. Various compounds have been screened over the years, and although some good hits have been reported, an efficient, selective, and cost-effective alternative is yet to be found. Technological advances have allowed target-based drug discovery to be widely applied in searching for drug candidates for several diseases. Recently, studies have started to explore this approach for discovering anti-*T. vaginalis* compounds. Also, studies investigating the mechanisms of action of promising drug candidates have been encouraged. In this scenario, studies characterizing and validating *T. vaginalis* drug targets are likely to become of interest, and although many virulence factors and potential drug targets have been described, only a few have recently started to be explored. Tapping into target-based drug discovery, target deconvolution, and approaches combining phenotype and target-based approaches might be the next challenges researchers will face in the search for alternative treatments for trichomoniasis.

## Supplementary Information

Below is the link to the electronic supplementary material.

ESM 1 (DOCX 18.4 KB)

## Data Availability

The datasets generated during and/or analyzed during the current study are available from the corresponding author upon reasonable request.

## Abbreviations

APs: Adhesin proteins
ATP: Adenosine triphosphate
CLS: Cyst-like structures
CPs: Cysteine proteases
*Hs*TIM: *Homo sapiens* Triosephosphate isomerase
IC_50_: Half maximal inhibitory concentration
MGL: Methionine gamma-lyase
MIC: Minimum inhibitory concentration
MTZ: Metronidazole
PFOR: Pyruvate-ferredoxin oxidoreductase
SEC: Secnidazole
STI: Sexually transmitted infection
TIM: Triosephosphate isomerase
TNZ: Tinidazole
*Tv*LDH: *Trichomonas vaginalis* Lactate dehydrogenase
*Tv*LG: *T. vaginalis* Surface lipoglycan
*Tv*MGL: *Trichomonas vaginalis* Methionine gamma-lyase
*Tv*TIM: *Trichomonas vaginalis* Triosephosphate isomerase
*Tv*TrxR: *Trichomonas vaginalis* Thioredoxin reductase
