# Supplementary material for: Trichomonas vaginalis Drug Targets and Their Role in Drug Discovery and Development
Source: Pharm Res. 2026 May 5;43(5):1357–78. doi: 10.1007/s11095-026-04098-0 (PMC13269401; doi:10.1007/s11095-026-04098-0)
Supplement: Supplementary file 1 — (DOCX 18.4 KB) [file 11095_2026_4098_MOESM1_ESM.docx]

**Table S1.** Criteria and rationale used for the qualitative scoring of *T. vaginalis* drug targets. Each criterion was scored from 1 to 3, with higher scores reflecting more favorable characteristics for drug development. This framework is intended to provide a structured and transparent qualitative comparative tool for target interpretation and prioritization, but does not represent a definitive or exhaustive assessment of target suitability.

| Parameters | Score | Definition | Rationale for scoring |
| --- | --- | --- | --- |
| Validation level | 1 | *In silico* evidence only | Lowest level of validation; hypothesis-generating |
|  | 2 | *In vitro* evidence (biochemical and/or cell-based) | Demonstrates biological relevance under controlled conditions |
|  | 3 | *In vivo* evidence and/or multi-level validation | Strongest support for physiological relevance and target essentiality |
| Druggability | 1 | Unclear binding site; complex system | Uncertain tractability |
|  | 2 | Partially characterized binding sites; moderately tractable | Potential for optimization, but may require careful design |
|  | 3 | Well-defined enzyme target with known inhibitors | Favors selective targeting and safer therapeutic profiles |
| Selectivity | 1 | Low selectivity/High similarity to human homologs | Increased risk of host toxicity |
|  | 2 | Moderate selectivity/average similarity to human homologs | Potential for optimization, but may require careful design |
|  | 3 | High parasite specificity/ low similarity to human homologs | Favors selective targeting and safer therapeutic profiles |
| Chemical matter availability | 1 | No known inhibitors | Early-stage target with limited translational readiness |
|  | 2 | Tool compounds or moderate inhibitors available | Enables preliminary validation and optimization |
|  | 3 | Potent/validated inhibitors or repurposed drugs available | Facilitates rapid progression toward preclinical/clinical studies |
| Resistance liability* | 1 | High risk (redundancy, isoforms, compensatory pathways) | Likely to enable rapid emergence of resistance |
|  | 2 | Moderate risk | Some potential for adaptation, but manageable |
|  | 3 | Low risk (essential, non-redundant pathways) | Reduced the likelihood of resistance development |

*Note: Resistance liability is inversely scored.
